# Evaluating Tech Neck: A Pilot Study Using a Self-Developed Questionnaire on Symptoms, Posture, and Preventive Measures

**DOI:** 10.3390/children12010102

**Published:** 2025-01-17

**Authors:** Brigitte Osser, Csongor Toth, Carmen Delia Nistor-Cseppento, Gyongyi Osser, Caius Calin Miuța, Iosif Ilia, Diana Carina Iovanovici, Cristina Aur, Laura Ioana Bondar

**Affiliations:** 1 Doctoral School of Biomedical Sciences, University of Oradea, 410087 Oradea, Romania; brigitte.osser@uav.ro (B.O.); csongor.toth@uav.ro (C.T.); dcseppento@uoradea.ro (C.D.N.-C.); bondar.lauraioana@student.uoradea.ro (L.I.B.); 2 Faculty of Physical Education and Sport, “Aurel Vlaicu” University of Arad, 310130 Arad, Romania; gyongyi.osser@uav.ro (G.O.); iosif.ilia@uav.ro (I.I.); 3 Department of Psycho-Neuroscience and Recovery, Faculty of Medicine and Pharmacy, University of Oradea, 410073 Oradea, Romania; 4 Institute of Cardiovascular Diseases Timișoara, Gheorghe Adam Street, No. 13A, 300310 Timișoara, Romania; iovanovici.dianacarina@student.uoradea.ro; 5 Department of Surgical Disciplines, Faculty of Medicine and Pharmacy, University of Oradea, 410073 Oradea, Romania; d.aur@uoradea.ro; 6 Department of Biology and Life Sciences, Faculty of Medicine, “Vasile Goldiș” Western University of Arad, 310048 Arad, Romania

**Keywords:** break frequency, ergonomic tools, measures taken, neck pain, posture awareness, symptom intensity, tech neck, wrist pain

## Abstract

Background/Objectives: Musculoskeletal symptoms, such as neck pain, back pain, and eye strain, are prevalent in modern work environments, particularly among individuals engaged in prolonged sitting and repetitive tasks. This study aimed to explore the prevalence of these symptoms and the impact of ergonomic tool usage, break frequency, posture awareness, and proactive measures on symptom intensity and daily functioning. Methods: This cross-sectional pilot study used a self-developed questionnaire to assess the prevalence and intensity of musculoskeletal symptoms among participants aged 6–18 years. The survey collected data on ergonomic tool usage, break frequency, posture awareness, and proactive measures (such as taking breaks and seeking professional help). Symptom intensity was assessed across common musculoskeletal issues. Internal consistency was assessed using Cronbach’s Alpha. Statistical analyses, including descriptive statistics, Chi-squared tests, ANOVA, and Spearman’s correlation, were used to identify significant correlations and associations between variables. Results: A significant proportion of participants reported moderate to severe symptoms, including neck pain, back pain, and eye strain. Participants who used ergonomic tools reported significantly lower symptom intensity (<0.001). A positive correlation was found between fewer breaks and higher symptom intensity (r = 0.908, *p* < 0.001). Those with higher posture awareness reported lower symptom intensity (*p* < 0.001). Proactive measures, including regular breaks and ergonomic interventions, were perceived as effective strategies for managing symptoms. Increased symptom intensity was associated with reduced ability to perform daily activities (*p* < 0.001). Conclusions: The study highlights the significant role of ergonomic tools, posture awareness, and regular breaks in mitigating musculoskeletal symptoms. Proactive measures, including structured interventions, can effectively reduce symptom intensity and improve daily functioning. These findings suggest that workplace health strategies focusing on ergonomics and posture education are crucial for reducing the impact of musculoskeletal discomfort on employee well-being and productivity.

## 1. Introduction

In the modern digital age, the widespread use of smartphones, laptops, and tablets has led to a rise in musculoskeletal discomfort, commonly referred to as “tech neck” [1,2]. This condition manifests as pain and stiffness in the neck, shoulders, and upper back, along with symptoms such as headaches, eye strain, and wrist discomfort [3,4]. It is typically linked to prolonged poor posture during device use, affecting a broad range of individuals, including office workers, students, remote workers, and general consumers [5,6]. Given technology’s integral role in daily life, addressing tech neck is crucial for public health, as it can result in long-term discomfort and hinder daily activities [7,8].

The literature identifies several factors contributing to tech neck, such as improper posture, infrequent breaks, and inadequate ergonomic practices [9,10]. While studies have explored individual factors like posture, ergonomic tools, and break frequency, few have examined their combined impact on symptom severity. Furthermore, much of the research has focused on specific populations, such as office workers, leaving a gap in understanding how tech neck affects more diverse groups, including students [11,12,13].

There is also a need for a standardized tool to assess tech neck symptoms and their impact on daily activities. While some generalized questionnaires measure musculoskeletal discomfort, few are designed specifically for tech neck, particularly to account for variations in symptom severity due to ergonomic factors, posture awareness, and preventive measures like breaks and ergonomic tools [14,15,16].

In addition to device-related factors, other lifestyle elements, such as extracurricular activities, may also influence musculoskeletal health. Activities like sports or playing musical instruments often involve repetitive motions or asymmetrical postures, which, if not properly managed or coached, can lead to strain and discomfort. For instance, sports such as tennis or activities like playing the violin can create uneven physical demands on the body, potentially compounding the effects of poor posture during device use [17,18,19,20,21]. While this study primarily focuses on device-related factors, considering the role of such activities is essential to comprehensively address musculoskeletal discomfort. These interactions could be explored further in future studies to provide more targeted intervention strategies for diverse populations. Building on this broader understanding of musculoskeletal discomfort, this study aims to address gaps in the assessment and management of tech neck symptoms.

The rationale for this study is to fill these gaps by developing a self-administered pilot questionnaire tailored to tech neck. The tool will assess symptoms, their severity, and the effectiveness of preventive and corrective measures such as posture adjustments, ergonomic tool usage, and break frequency. By providing a more accurate means of assessing tech neck’s impact, this research will offer valuable insights into the effectiveness of various interventions.

Ultimately, the primary aim is to develop and test a comprehensive tool for evaluating tech neck symptoms and associated factors, focusing on the relationships between posture, ergonomic practices, break frequency, and symptom severity. The findings from this research will inform future research and practical strategies for managing and preventing tech neck symptoms.

## 2. Materials and Methods

### 2.1. Study Design and Setting

This study adopts a cross-sectional design to evaluate the prevalence and severity of tech neck symptoms among students. Data collection was conducted through a self-administered pilot questionnaire, which was distributed to participants at Vinga Technological High School, located in Arad, Romania. The study period spans from October 2023 to October 2024.

### 2.2. Study Population

The study population consists of 222 students from various educational levels, including primary school (Grades 1–4), middle school (Grades 5–8), and high school (Grades 9–12). Participants were selected from the school community to assess tech neck symptoms, posture, and the use of preventive measures across different age groups and academic levels.

### 2.3. Eligibility Criteria

#### 2.3.1. Inclusion Criteria

The following criteria were used to determine student eligibility for participation in the study:

- Age: Students aged 6 to 18 years, enrolled in primary, middle, or high school levels at Vinga Technological High School during the study period;
- Gender: Both male and female students were included in the study to ensure a diverse representation of experiences with tech neck symptoms;
- Willingness to Participate: Students who voluntarily agreed to participate in the study and complete the questionnaire regarding their device usage and symptoms;
- Informed Consent: All participants provided informed consent, confirming their understanding of the study’s purpose, procedures, and potential risks;
- Parental Consent: Parental consent was obtained for all students under the age of 18, ensuring that parents or guardians were fully informed about the study;
- Comprehension and Assistance: Students were required to understand and be able to complete the questionnaire. To ensure clarity and accuracy, the researcher was present during the questionnaire administration to provide guidance and clarification. Parents or guardians were also encouraged to assist younger participants as needed.

#### 2.3.2. Exclusion Criteria

Participants who did not meet the following conditions were excluded from the study:

- Pre-existing Musculoskeletal Conditions: Students with known chronic musculoskeletal disorders or other significant health conditions that could interfere with the assessment of tech neck symptoms were excluded from the study;
- Inability to Complete the Questionnaire: Students who were unable to understand or complete the questionnaire due to language barriers, cognitive impairments, or other limitations were excluded;
- Non-voluntary Participation: Students who did not provide informed consent to participate or whose parents did not grant consent for their involvement in the study were excluded;
- Unavailability for Data Collection: Students who were absent during the data collection period were excluded from the study.

**Figure 1:**
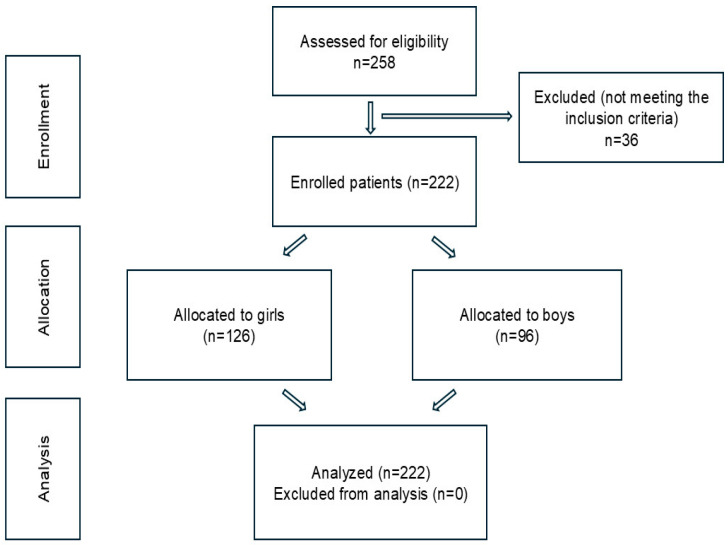
Flow chart of participant enrollment and analysis.

The flow of participants through the study is summarized in Figure 1. A total of 258 students were assessed for eligibility based on the inclusion and exclusion criteria. Out of these, 36 students were excluded for pre-existing musculoskeletal conditions (12 students), inability to complete the questionnaire (10 students), and lack of informed or parental consent (14 students). Following these exclusions, a total of 222 students were enrolled in the study, comprising 126 girls and 96 boys. Data from all 222 participants were successfully collected and included in the analysis, with no exclusions during the analysis phase. The complete inclusion of all enrolled participants in the analysis ensures that the results accurately reflect the study population.

### 2.4. Data Collection Instruments

The primary tool for data collection was the self-developed “Tech Neck” Syndrome Questionnaire, designed to assess the presence of tech neck symptoms, the frequency of device usage, posture awareness, ergonomic behaviors, and preventive measures [Supplementary Materials, File S1]. The questionnaire was divided into seven sections.

#### 2.4.1. Questionnaire Sections

1. Demographic Data: Includes age, gender, school level, and physical activity level;
2. Device Usage: Questions regarding the types of electronic devices used daily, duration of device usage, sitting posture while using devices, and break frequency;
3. Symptoms and Associated Discomfort: Assesses the frequency and severity of symptoms like neck pain, shoulder pain, back pain, headaches, numbness in hands or arms, eye strain, and wrist pain;
4. Ergonomics: Investigates the use of ergonomic tools such as laptop stands, special chairs, or monitor stands;
5. Impact on Daily Life: Evaluates how neck or back problems impact daily activities and whether students skip activities due to discomfort;
6. Measures Taken: Assesses coping mechanisms for tech neck symptoms, such as taking breaks, stretching, using ergonomic tools, or seeking professional help;
7. Posture Awareness: Evaluates the participant’s awareness of posture when using electronic devices.

#### 2.4.2. Protocol Validation of the Self-Developed Questionnaire

The “Tech Neck” Syndrome Questionnaire underwent rigorous validation procedures to ensure its reliability and accuracy in assessing tech neck symptoms and associated factors [Supplementary Materials, File S1]. The validation process involved two key components: internal consistency and comparative validity.

1. Internal Consistency

To assess the internal consistency of the questionnaire, Cronbach’s Alpha (α) was calculated across all items. The resulting Cronbach’s α value demonstrated excellent reliability, indicating that the questionnaire is internally consistent. This high level of internal consistency ensures that the items are well-correlated and contribute meaningfully to the construct of tech neck symptoms. A value of 0.9 or higher was considered indicative of excellent reliability, confirming that the questionnaire effectively measures the intended aspects of tech neck, including symptoms such as neck pain, back pain, and wrist discomfort.

2. Comparative Validity with Other Validated Questionnaires

To establish the comparative validity of the “Tech Neck” Syndrome Questionnaire, its results were compared with two widely used, validated tools in Romania that measure musculoskeletal discomfort and ergonomic-related symptoms:

- The Nordic Musculoskeletal Questionnaire (NMQ): This questionnaire is commonly used in Romania to assess musculoskeletal symptoms, particularly in relation to neck, back, and shoulder pain. It has been extensively validated and applied across different regions, including Arad. The NMQ provides a valuable benchmark for comparing the frequency and intensity of musculoskeletal discomfort, which aligns closely with the symptoms assessed by the “Tech Neck” Syndrome Questionnaire;
- The Visual Analog Scale (VAS) for Pain: The VAS is a standard tool widely used in Romania to evaluate pain intensity. It is validated for use in clinical and research settings and offers a measure of pain severity, which is highly relevant to the symptoms reported in tech neck cases, such as neck, back, and wrist pain. By comparing the results of the “Tech Neck” Syndrome Questionnaire with the VAS, the study ensures that the pain assessments are consistent with widely accepted measures in the field.

The comparative validity of the self-developed questionnaire was established through the alignment of its findings with those of the NMQ and VAS, confirming that the “Tech Neck” Syndrome Questionnaire provides reliable and valid results in the context of musculoskeletal and ergonomic discomfort in Romania.

This dual validation approach—both internal consistency and comparative validity—ensures that the “Tech Neck” Syndrome Questionnaire is a robust tool for assessing tech neck symptoms in adolescents, with reliable and valid data that can be compared to other established measures in the field.

### 2.5. Ethical Considerations

The study was approved by the Ethical Committee of Vinga Technological High School under approval number 1436/2/16 October 2023. Ethical considerations included informed consent obtained from both participants and their guardians, ensuring that participation was voluntary and confidential. The study adheres to ethical principles of research, including respect for privacy, anonymity, and the right to withdraw from the study at any point.

### 2.6. Data Analysis

Data were analyzed using JASP 0.19.2 (University of Amsterdam, Amsterdam, The Netherlands). Descriptive statistics were initially applied to summarize demographic information, including age, gender, and school level, as well as device usage patterns. Frequency counts and percentages were calculated for the severity of symptoms (neck pain, shoulder pain, back pain, eye strain, etc.) to assess the distribution and frequency of discomfort experienced by participants.

To examine the relationships between posture awareness, device usage, ergonomic practices, and symptom severity, Spearman’s correlation was performed to assess the strength and direction of the relationships between continuous variables. Chi-squared tests were employed to evaluate associations between categorical variables, such as device usage habits and the presence of symptoms.

The relationship between these variables was further explored through regression analyses to determine potential predictive factors for symptom severity. The significance level was set at *p* < 0.05, indicating statistical significance. This allowed for the identification of any meaningful associations between the factors examined in this study.

### 2.7. Hypotheses of the Study

This section outlines the hypotheses formulated to guide the investigation of the relationship between ergonomic practices, posture awareness, symptom intensity, and their impact on daily activities. The study aims to identify patterns and correlations among various factors contributing to musculoskeletal discomfort and associated symptoms. By testing these hypotheses, the study seeks to provide evidence-based insights into the effectiveness of ergonomic interventions and behavioral strategies in reducing symptom severity and enhancing daily functioning. The hypotheses are as follows:

1. There will be a significant proportion of participants who report moderate to severe symptoms of neck pain, shoulder pain, back pain, headaches, numbness, eye strain, and wrist pain;
2. Participants who use ergonomic tools will report significantly lower symptom intensity compared to those who do not use ergonomic tools;
3. There will be a significant positive correlation between fewer breaks and higher symptom intensity among participants;
4. Individuals with higher posture awareness will report significantly lower symptom intensity compared to those with lower posture awareness;
5. Participants who take proactive measures, such as taking breaks, using ergonomic tools, and seeking professional help, will perceive these actions as more effective in alleviating symptoms than those who take less structured actions;
6. The intensity of symptoms (neck pain, shoulder pain, back pain, headaches, numbness, eye strain, and wrist pain) will be significantly associated with a reduction in the ability to perform daily activities;
7. Individuals who avoid activities due to their symptoms will report significantly higher symptom intensity compared to those who do not avoid activities.

## 3. Results

The following section presents the key findings of the study, highlighting the statistical analyses performed to evaluate the relationships between ergonomic practices, symptom intensity, and their impact on daily activities. Descriptive and inferential statistics, including Chi-square tests, ANOVA, Spearman correlations, and reliability analyses, are utilized to provide a comprehensive understanding of the data. These results address the study’s objectives, offering insights into how ergonomic tool usage, posture awareness, break frequency, and other factors influence symptom severity and functional outcomes.

### 3.1. Demographic Profile

#### 3.1.1. Age Distribution of Participants

Table 1 presents the descriptive statistics for the Age variable based on responses from 222 participants, with no missing data. The median age of 14.500 years indicates that half of the participants are younger than 14.5 years, while the other half are older. The mean age of 13.396 years is slightly lower than the median, reflecting a slight skew toward younger participants.

The standard deviation of 3.851 shows moderate variability in the ages of participants, suggesting a spread around the mean. The range of 12 years indicates the difference between the youngest and oldest participants, highlighting the diversity of ages included in the sample.

This table provides a clear summary of the age distribution among the respondents, which is essential for understanding the demographic composition of the study group.

#### 3.1.2. Demographic Characteristics of Study Participants

The data summarized in Table 2 provides a comprehensive view of the participants’ demographics, with insights into gender, educational levels, and physical activity patterns, which are critical for understanding lifestyle factors that may influence the occurrence of “tech neck” syndrome.

- Gender: Among the 222 participants, 43.24% (96) were boys, and 56.76% (126) were girls, showing a slightly higher proportion of female respondents.
- School Level: Participants were drawn from three school levels: 20.27% (45) were in primary school, 30.18% (67) in middle school, and 49.55% (110) in high school. This distribution indicates that nearly half of the sample comprised older students.
- Physical Activity: Responses to the question on activity levels revealed that 50.00% (111) of participants identified as sedentary, spending most of their time sitting or relaxing. 26.57% (59) were moderately active, engaging in occasional physical activity, while 23.42% (52) were active, participating frequently in sports or exercise.
- Missing Data: No missing data were reported, confirming that all participants provided complete responses for these demographic variables.

### 3.2. Prevalence of Device Use Among Participants

Table 3 presents the distribution of device ownership among the respondents. The vast majority of participants (96.40%) reported owning a smartphone, while only 3.60% did not. Regarding tablet ownership, 34.69% of respondents reported owning a tablet, while 65.31% did not. For laptops, 60.81% of participants indicated they owned one, while 39.19% did not. Lastly, 40.54% of respondents reported using a computer, with 59.46% not owning one. These results highlight the widespread use of smartphones and laptops, while tablet and desktop computer ownership are less common among the participants.

### 3.3. Device Usage and Posture Impact

#### 3.3.1. Correlation Between Device Usage Time and Symptom Intensity

Table 4 presents the results of the Spearman’s rank correlation between Device Usage Time and Symptom Intensity.

- The Spearman’s rho value of 0.908 indicates a strong positive correlation between Device Usage Time and Symptom Intensity. This suggests that as device usage time increases, symptom intensity also increases substantially;
- The *p*-value of <0.001 is less than 0.05, indicating that this correlation is statistically significant. This shows that the observed strong positive correlation is unlikely to have occurred by chance.

In summary, Device Usage Time and Symptom Intensity are statistically significantly correlated, with a strong correlation.

#### 3.3.2. The Linear Regression Analysis: Device Usage Time and Symptom Intensity

A linear regression analysis was conducted to explore the relationship between device usage time and symptom intensity. The predictor variable was device usage time, and the outcome variable was symptom intensity.

Table 5 presents the results of the linear regression analysis examining the relationship between device usage time and symptom intensity. Two models are shown: the intercept-only model (M_0_) and the model that includes device usage time (M_1_).

- The intercept-only model (M_0_), which does not include any predictors, shows no explanatory power. The R^2^ value is 0.000, indicating that this model does not account for any variance in symptom intensity. The RMSE (Root Mean Square Error) of 2.181 also suggests that the predictions from this model are highly inaccurate;
- The model with device usage time (M_1_) shows a dramatic improvement. The R-value of 0.908 indicates a very strong relationship between device usage time and symptom intensity. The R^2^ value of 0.824 means that device usage time explains 82.4% of the variance in symptom intensity. This is a very strong predictor of symptom severity. The RMSE of 0.917 indicates that the model’s predictions are much more accurate than the intercept-only model.

Table 6 presents the ANOVA results for the regression analysis, which assesses the overall significance of the model. The F value of 1029.845 and a *p*-value of <0.001 indicate that the regression model with device usage time is statistically significant. This means the model explains a significant portion of the variance in symptom intensity, making it a valid predictor of symptom severity.

Table 7 displays the coefficients for the regression model. The coefficient for device usage time is 1.890, with a standard error of 0.059. The standardized coefficient is 0.908, suggesting that for each one-unit increase in device usage time, symptom intensity increases by 1.890 units on average. The t-value of 32.091 and the *p*-value of <0.001 confirm that the relationship between device usage time and symptom intensity is statistically significant.

The results of the regression analysis demonstrate a robust and statistically significant relationship between device usage time and symptom intensity. As the duration of device use increases, symptom intensity also increases.

#### 3.3.3. Association Between Posture and Symptom Occurrence

Table 8 presents the results of the Chi-squared tests assessing the association between posture and various symptoms. In this study, “posture” was assessed based on the participants’ responses to the question: “How do you usually sit or lie when using your devices?” Participants selected their typical posture, such as sitting at a desk, lying in bed, standing, sitting on a couch, or other positions. The tests revealed significant associations between posture and all the symptoms tested, with all *p*-values being <0.001, specifically the following:

- Neck Pain (χ^2^ = 267.915, df = 16, *p* < 0.001);
- Shoulder Pain (χ^2^ = 299.668, df = 16, *p* < 0.001);
- Back Pain (χ^2^ = 256.901, df = 16, *p* < 0.001);
- Headaches (χ^2^ = 251.836, df = 16, *p* < 0.001);
- Numbness (χ^2^ = 286.069, df = 16, *p* < 0.001);
- Eye Strain (χ^2^ = 284.561, df = 16, *p* < 0.001);
- Wrist Pain (χ^2^ = 251.436, df = 16, *p* < 0.001);
- Symptom Intensity (χ^2^ = 269.741, df = 32, *p* < 0.001).

Since the *p*-values for all the tests are <0.001, these results indicate a statistically significant association between posture and each symptom. This suggests that posture is an important factor in the occurrence and intensity of symptoms such as neck pain, back pain, headaches, and others, emphasizing the role of postural habits in musculoskeletal health.

### 3.4. Symptoms and Ergonomics

#### 3.4.1. Symptom Prevalence

Table 9 presents the frequencies and percentages of various symptoms, including neck pain, shoulder pain, back pain, headaches, numbness, eye strain, wrist pain, and symptom intensity, as reported by the sample of 222 participants. For each symptom, participants were asked to rate the severity on a scale from 1 (very mild) to 5 (very bad) or for symptom intensity, from 1 (very mild) to 10 (very bad).

- Back Pain: The majority of participants reported moderate to severe neck pain, with 38.74% (86 individuals) indicating the most severe level of pain (level 5). 31.53% (70 individuals) reported level 4 pain, while 17.12% (38 individuals) reported level 3, suggesting a notable presence of neck pain in the sample. A smaller proportion reported lower levels of pain, with 10.81% (24 individuals) at level 2 and only 1.80% (4 individuals) at level 1, indicating mild discomfort;
- Neck Pain: The majority of participants reported moderate to severe neck pain, with 38.74% (86 individuals) indicating the most severe level of pain (level 5). 31.53% (70 individuals) reported level 4 pain, while 17.12% (38 individuals) reported level 3, suggesting a notable presence of neck pain in the sample. A smaller proportion reported lower levels of pain, with 10.81% (24 individuals) at level 2 and only 1.80% (4 individuals) at level 1, indicating mild discomfort;
- Shoulder Pain: Similarly, shoulder pain was predominantly reported at higher levels, with 41.89% (93 individuals) experiencing severe pain (level 5). Moderate levels of shoulder pain were also common, with 31.08% (69 individuals) reporting level 4 and 15.32% (34 individuals) at level 3. Only 9.46% (21 individuals) reported moderate shoulder pain at level 2, and 2.25% (5 individuals) reported mild discomfort at level 1;
- Back Pain: Back pain was reported most frequently at the highest severity level, with 42.79% (95 individuals) indicating severe pain (level 5). 28.38% (63 individuals) reported level 4 pain, and 16.67% (37 individuals) reported moderate pain (level 3). Fewer participants reported mild back pain, with 10.36% (23 individuals) at level 2 and only 1.80% (4 individuals) at level 1;
- Headaches: The most common severity level for headaches was level 5, with 40.09% (89 individuals) reporting very severe headaches. 34.23% (76 individuals) indicated level 4 pain, while moderate headaches at level 3 were reported by 14.87% (33 individuals). 9.91% (22 individuals) reported mild headaches at level 2, and only 0.90% (2 individuals) experienced very mild headaches at level 1;
- Numbness: A significant proportion of participants experienced severe numbness, with 37.84% (84 individuals) reporting the highest severity level (level 5). 34.68% (77 individuals) reported level 4 numbness, while 14.87% (33 individuals) reported moderate numbness at level 3. 10.36% (23 individuals) reported mild numbness at level 2, and 2.25% (5 individuals) reported very mild numbness at level 1. These findings may reflect high device usage among this age group, leading to potential strain in the hands, arms, or neck. As the data were self-reported, they may differ from clinically assessed prevalence rates;
- Eye Strain: Eye strain was most prevalent at the highest severity level, with 42.79% (95 individuals) reporting severe symptoms (level 5). 27.93% (62 individuals) indicated level 4 eye strain, and 18.02% (40 individuals) reported moderate strain (level 3). 9.01% (20 individuals) reported mild eye strain at level 2, and 2.25% (5 individuals) indicated very mild symptoms at level 1;
- Wrist Pain: Wrist pain also followed a similar trend, with 37.84% (84 individuals) reporting the most severe level (level 5). 34.68% (77 individuals) reported level 4 wrist pain, and 15.77% (35 individuals) experienced moderate pain at level 3. 9.01% (20 individuals) reported mild wrist pain at level 2, and 2.70% (6 individuals) indicated very mild symptoms at level 1;
- Symptom intensity: Refers to the overall severity of the symptoms experienced by participants, ranging from very mild (1) to very bad (10). The table provides the distribution of how participants rated their symptom intensity.
  ○ Severe Symptoms: The highest symptom intensities (levels 9 and 10) were reported by a substantial proportion of the sample. Specifically, 21.17% (47 individuals) rated their symptom intensity as 10 (very bad), and 22.07% (49 individuals) rated it at 8. Additionally, 18.92% (42 individuals) reported a severity level of 9, indicating that nearly 62% of participants experienced either severe or very severe symptoms.
  ○ Moderate Symptoms: A moderate level of symptom intensity (levels 6 and 7) was reported by 7.21% (16 individuals) for each of these levels. This suggests that while the majority of participants reported more severe symptoms, a portion still experienced moderate intensity;
  ○ Mild Symptoms: Lower levels of symptom intensity (levels 1 to 5) were less common, with 9.91% (22 individuals) reporting level 5, 8.11% (18 individuals) at level 4, and 4.95% (11 individuals) at level 3. Very mild symptoms (level 1) were not reported, which suggests that participants did not consider their symptoms as trivial or negligible.

#### 3.4.2. Association Between Ergonomic Tool Usage and Symptom Intensity

Table 10 shows the distribution of symptom intensity levels across the five ergonomic tool usage categories. A Chi-squared test revealed a statistically significant association between ergonomic tool usage and symptom intensity (χ^2^ = 306.803, df = 32, *p* < 0.001). This suggests that the use of ergonomic tools is significantly associated with the severity of symptoms, with varying levels of symptom intensity observed across different usage groups.

Table 11 shows the results of the ANOVA, which indicate significant differences in symptom intensity across the five ergonomic tool usage groups (F (4, 217) = 115.881, *p* < 0.001). This suggests that ergonomic tool usage has a statistically significant effect on the severity of symptoms, with varying levels of symptom intensity observed across the different tool usage groups.

#### 3.4.3. Association Between Breaks and Symptom Intensity

Table 12 presents the Spearman’s rank correlation between Symptom Intensity and Breaks. The analysis yielded a strong positive correlation (Spearman’s rho = 0.740, *p* < 0.001). This suggests that as the frequency of breaks decreases (i.e., moving from every 30 min to rarely or never), the symptom intensity tends to increase. These results indicate a positive relationship between fewer breaks and higher symptom intensity.

### 3.5. Awareness and Behavioral Changes

#### 3.5.1. Association Between Posture Awareness and Symptom Intensity

Table 13 presents the Chi-square test results examining the relationship between posture awareness and symptom intensity. The analysis revealed a statistically significant association (χ^2^ = 180.547, df = 24, *p* < 0.001). This suggests that individuals with varying levels of posture awareness exhibit distinct patterns of symptom intensity severity. The results indicate that those who are more aware of their posture tend to report less severe symptoms compared to individuals who rarely or never consider their posture.

#### 3.5.2. Association Between Measures Taken and Effectiveness

Table 14 presents the results of a Chi-square test examining the relationship between measures taken to alleviate symptoms (e.g., taking breaks, stretching, using ergonomic tools, or seeking professional help) and their perceived effectiveness. The test revealed a statistically significant association between the measures taken and their effectiveness (χ^2^ = 343.855, df = 16, *p* < 0.001). This indicates that different measures vary significantly in their perceived ability to relieve symptoms. For example, more proactive approaches, such as getting a massage or using ergonomic tools, may be associated with higher levels of perceived effectiveness, while less proactive approaches are likely associated with lower levels of effectiveness.

These findings suggest that individuals who engage in targeted actions to address discomfort, such as stretching, using specialized tools, or seeking professional treatment, tend to perceive greater relief from their symptoms compared to those who take no measures or rely solely on less structured actions like occasional breaks.

### 3.6. Impact on Daily Activities

#### 3.6.1. Impact of Symptoms Intensity on Daily Activities

The Chi-square test results presented in Table 15 show the relationship between various symptoms and their potential impact on activities. All the symptoms listed have a *p*-value of less than 0.001, which is highly statistically significant.

- Neck Pain (χ^2^ = 232.260, df = 16, *p* < 0.001);
- Shoulder Pain (χ^2^ = 254.075, df = 16, *p* < 0.001);
- Back Pain (χ^2^ = 261.429, df = 16, *p* < 0.001);
- Headaches (χ^2^ = 246.721, df = 16, *p* < 0.001);
- Numbness (χ^2^ = 244.467, df = 16, *p* < 0.001);
- Eye Strain (χ^2^ = 281.664, df = 16, *p* < 0.001);
- Wrist Pain (χ^2^ = 249.263, df = 16, *p* < 0.001);
- Symptom Intensity (χ^2^ = 269.741, df = 32, *p* < 0.001).

These results indicate that the severity of neck pain, shoulder pain, back pain, headaches, numbness, eye strain, and wrist pain are significantly associated with changes in the ability to perform daily activities. Individuals who experience these symptoms are more likely to report varying degrees of activity impairment.

#### 3.6.2. Relationship Between Activity Avoidance and Symptom Intensity

Table 16 presents the results of an ANOVA comparing symptom intensity between individuals who avoid activities due to their symptoms and those who do not. The analysis revealed a statistically significant difference in symptom intensity between the two groups (F (1, 220) = 280.048, *p* < 0.001). This suggests that individuals who avoid activities because of the severity of their symptoms experience significantly higher symptom intensity than those who do not avoid activities. These findings underscore the impact of symptom severity on daily functioning, as more intense symptoms appear to limit the ability to engage in physical activities.

### 3.7. Internal Consistency and Reliability of the Questionnaire

Table 17 presents the Cronbach’s α coefficient for the overall scale, as well as the Cronbach’s α of the item dropped for each individual item. Cronbach’s α is used to assess the internal consistency of the questionnaire, with values closer to 1.0 indicating a high level of reliability. The overall Cronbach’s α for the questionnaire is 0.965, with a 95% Confidence Interval (CI) ranging from 0.961 to 0.968, suggesting excellent internal consistency across the entire set of items. This indicates that the items collectively measure the intended construct in a highly reliable manner.

Table 18 presents the Cronbach’s α if item dropped values for each item in the questionnaire, alongside the corresponding 95% CI. These values indicate the internal consistency of the scale when each item is removed individually. The Cronbach’s α values for all items range from 0.960 to 0.968, with the lower bound of the 95% CI ranging from 0.957 to 0.965 and the upper bound ranging from 0.960 to 0.972. These values suggest that removing any single item from the scale does not significantly alter its overall reliability. In particular, the items related to Laptop, Smartphone, and Tablet show slightly higher α values (ranging from 0.967 to 0.968), indicating that these items contribute strongly to the overall internal consistency of the scale.

Overall, the high Cronbach’s α values, consistently above 0.960, indicate that each item contributes well to the scale’s reliability, and there is no indication that omitting any individual item would substantially improve the internal consistency of the questionnaire. This further supports the robustness of the measurement tool.

## 4. Discussion

This study aimed to explore the prevalence of musculoskeletal symptoms among participants, particularly focusing on the intensity of these symptoms in relation to ergonomic tool usage, break frequency, posture awareness, and proactive measures. The findings of this study provide important insights into the relationship between these factors and symptom intensity, with implications for the health and well-being of individuals.

### 4.1. Interpretation of Symptom Prevalence

This study’s findings suggest that a significant proportion of participants reported moderate to severe symptoms of neck pain, shoulder pain, back pain, headaches, numbness, eye strain, and wrist pain. This supports previous studies indicating that musculoskeletal discomfort is highly prevalent in modern environments, particularly in those who engage in prolonged sitting or repetitive tasks [22,23]. These results underline the need for more effective interventions to address these widespread issues, which could not only improve individual comfort but also reduce the long-term health consequences of untreated symptoms.

Additionally, the findings may be influenced by differences in device access and usage patterns among participants, particularly between younger children and teenagers. For instance, younger participants (e.g., 8-year-olds) may have less frequent access to laptops or computers compared to older participants, leading to variations in posture and associated symptoms. These differences highlight the importance of considering age-related variations in device usage and their potential impact on musculoskeletal symptoms [24,25,26,27].

### 4.2. Ergonomic Tools and Symptom Intensity

Participants who used ergonomic tools reported significantly lower symptom intensity compared to those who did not use these tools. This finding is consistent with prior research that emphasizes the positive effects of ergonomic interventions on reducing physical discomfort [28,29,30]. Ergonomic tools, such as sit-stand desks, lumbar support chairs, and keyboard adjustments, can help reduce strain on the body by promoting better posture and providing physical support, thereby decreasing symptom intensity. These results highlight the importance of incorporating ergonomic tools to mitigate the risk of musculoskeletal disorders [31].

### 4.3. Break Frequency and Symptom Intensity

The study found a significant positive correlation between fewer breaks and higher symptom intensity, meaning that participants who took fewer breaks reported more intense symptoms. This result is consistent with previous studies showing that frequent breaks, particularly when standing or stretching, can help reduce the accumulation of physical strain [32,33]. In contrast, individuals who remain sedentary for extended periods are more likely to experience discomfort and fatigue. These findings reinforce the recommendation that individuals take regular breaks to reduce the risk of musculoskeletal symptoms and improve overall well-being [34,35].

### 4.4. Posture Awareness and Symptom Intensity

Individuals with higher posture awareness reported significantly lower symptom intensity than those with lower posture awareness. This result aligns with research indicating that poor posture is a major contributor to musculoskeletal pain, especially in the neck and lower back areas [36,37]. Greater awareness of posture allows individuals to make real-time adjustments, potentially reducing the strain on muscles and joints. This finding emphasizes the value of posture education and awareness as part of health promotion programs [38,39,40].

The widespread use of smartphones and laptops among participants may contribute to maladaptive postures, including accentuated kyphosis. Smartphone use, in particular, often involves a forward head posture with prolonged neck flexion, increasing strain on the cervical spine and upper back. This posture is associated with rounded shoulders, accentuated kyphosis, and musculoskeletal symptoms, as evidenced by previous studies [41,42,43].

Similarly, prolonged laptop use without proper ergonomic setups can exacerbate poor posture, such as slouching or hunching forward, due to screen positioning and the lack of appropriate back and arm support [44,45]. These postural habits are particularly concerning, given their association with increased symptom severity, as observed in this study. Addressing these postural issues through posture education and ergonomic interventions is essential for mitigating the long-term effects of device usage on musculoskeletal health.

### 4.5. Proactive Measures for Symptom Alleviation

Participants who adopted proactive measures, such as taking breaks, using ergonomic tools, and seeking professional help, perceived these actions as more effective in alleviating symptoms than those who took less structured actions. This suggests that structured and intentional approaches to symptom management are more likely to yield positive results. Previous studies have shown that a combination of physical activity, ergonomic interventions, and professional treatment can effectively alleviate musculoskeletal symptoms [46,47,48,49]. Our findings underscore the importance of encouraging individuals to adopt a multi-faceted approach to symptom management.

### 4.6. Impact of Symptoms on Daily Activities

The results indicate that higher symptom intensity was significantly associated with a reduction in the ability to perform daily activities. This finding corroborates previous research highlighting the functional impact of musculoskeletal disorders on daily living [50,51,52]. Symptoms of neck pain, shoulder pain, and other musculoskeletal issues can hinder physical tasks, leading to decreased productivity and lower quality of life [53,54]. These results emphasize the need for timely intervention and management strategies to prevent long-term disability.

### 4.7. Activity Avoidance and Symptom Intensity

Finally, participants who avoided activities due to their symptoms reported significantly higher symptom intensity compared to those who did not avoid activities. This suggests that activity avoidance may contribute to the worsening of symptoms, potentially leading to a cycle of physical inactivity and increased pain [55,56,57]. This is consistent with studies that suggest physical activity can play a crucial role in preventing and alleviating musculoskeletal symptoms [58,59,60]. Encouraging individuals to maintain a level of activity despite discomfort may help mitigate symptom progression and improve overall function.

### 4.8. Implications for Practice

The findings from this study have significant implications for health and individual well-being. The evidence supporting the use of ergonomic tools, regular breaks, posture awareness, and proactive symptom management suggests that employers should implement strategies to reduce musculoskeletal discomfort. These could include ergonomic workstation adjustments, scheduled breaks, and educational programs to promote posture awareness. Additionally, individuals should be encouraged to take a proactive role in managing their symptoms through these various approaches.

### 4.9. Future Research Directions

While this study provides valuable insights, further research is needed to explore the long-term effects of these interventions on symptom progression and the overall health of individuals. Longitudinal studies could examine whether the consistent use of ergonomic tools and adherence to break schedules lead to sustained improvements in symptom intensity and activity levels.

Additionally, more research is needed to explore the psychological factors that may influence symptom perception and the effectiveness of interventions. Investigating the role of mental health and coping strategies in musculoskeletal symptom management could provide a more comprehensive understanding of the issue.

The reported prevalence and severity of numbness in our study population were higher than expected based on typical epidemiological data. This discrepancy may be attributed to the self-reported nature of the questionnaire and the specific characteristics of the sample, which included frequent device users. Future research should incorporate objective measures and include more diverse populations to validate these findings and assess contributing factors such as device usage patterns, posture, and underlying health conditions.

Future studies could also analyze results separately for different age groups, particularly younger participants, to explore age-specific trends and tailor interventions accordingly. Differences in device access and usage patterns between age groups should also be investigated to better understand their influence on posture and musculoskeletal symptoms. For example, younger children may experience different patterns of strain compared to teenagers due to reduced exposure to prolonged device use. Such studies could help develop targeted ergonomic interventions and guidelines tailored to specific age groups.

Finally, future research should examine how age-specific differences influence behaviors such as activity avoidance and the adoption of proactive measures for symptom alleviation. Younger participants may exhibit different levels of symptom awareness and response strategies compared to older participants. Understanding these variations could help develop age-appropriate recommendations for symptom management and encourage effective behavioral interventions across all age groups.

### 4.10. Limitations of the Study

While this study provides valuable insights into the relationship between ergonomic tool usage, break frequency, posture awareness, and proactive measures in managing musculoskeletal symptoms, there are several limitations that must be acknowledged.

1. Cross-Sectional Design: The study employed a cross-sectional design, which means that data were collected at a single point in time. This limits the ability to draw conclusions about the causal relationships between variables. Longitudinal studies would be beneficial in determining the long-term effects of ergonomic interventions and proactive measures on musculoskeletal symptoms and daily activities;
2. Self-Reported Data: The data collected on symptom intensity, tool usage, break frequency, and posture awareness were based on self-reports, which are subject to biases such as social desirability bias and recall bias. Participants may have overestimated or underestimated their symptom severity or adherence to recommended practices. For example, younger participants may have differing levels of awareness or ability to accurately report their device usage and posture habits compared to older participants. Objective measures or observational data would provide more reliable insights into these factors;
3. Sample Size and Generalizability: The study’s sample size may not be large enough to fully represent diverse populations, especially across different age groups, occupations, or geographical locations. A larger and more diverse sample would enhance the generalizability of the findings and ensure that the results are applicable to various demographic groups. Additionally, the sample may have a higher proportion of individuals already experiencing musculoskeletal symptoms, which could affect the external validity of the findings;
4. Lack of Control for Confounding Variables: Although the study focused on specific factors like ergonomic tools, break frequency, and posture awareness, there may be other confounding variables that could influence symptom intensity and activity levels. For school-aged participants, factors such as academic stress, extended screen time for schoolwork, and developmental differences in posture and musculoskeletal systems may play a significant role. While this study captured general physical activity levels, it did not collect detailed data on specific extracurricular activities, such as sports or musical training, which are known to influence posture and musculoskeletal health. Future research should include these variables to better understand their interaction with sedentary behaviors and device usage patterns. Controlling for these factors in future studies would provide a clearer understanding of the relationship between the studied variables;
5. Limited Scope of Symptoms: The study focused on a specific set of symptoms (neck pain, shoulder pain, back pain, headaches, numbness, eye strain, and wrist pain), but other musculoskeletal disorders or symptoms not included in the study could also play a role in the overall symptom intensity and impact on daily activities. Expanding the scope to include a wider range of symptoms would provide a more comprehensive understanding of the issue;
6. Lack of Age-Specific Analysis: This study did not analyze age-specific trends in activity avoidance or the adoption of proactive measures for symptom alleviation. Age-related differences in symptom awareness, activity patterns, and management strategies could influence these behaviors, potentially impacting the findings. Future studies should categorize participants by age to better understand how symptom severity and management strategies vary across different developmental stages.

Despite these limitations, the study provides valuable insights into the factors influencing musculoskeletal symptoms and offers practical implications for improving health and individual well-being. Future research addressing these limitations can build on these findings to develop more effective and comprehensive strategies for preventing and managing musculoskeletal discomfort.

## 5. Conclusions

This study explored the relationships between ergonomic tool usage, break frequency, posture awareness, proactive measures, and the intensity of musculoskeletal symptoms in participants. The findings suggest that a significant proportion of individuals experience moderate to severe symptoms such as neck pain, shoulder pain, back pain, headaches, numbness, eye strain, and wrist pain. The use of ergonomic tools was found to be associated with a reduction in symptom intensity, supporting the importance of ergonomic interventions in alleviating discomfort. Additionally, higher posture awareness and the adoption of proactive measures, such as taking breaks and seeking professional help, were perceived as effective strategies for managing symptoms.

The study also revealed that a higher symptom intensity is associated with a reduced ability to perform daily activities, and individuals who avoid activities due to their symptoms report even higher levels of discomfort. These findings underline the significant impact that musculoskeletal symptoms can have on an individual’s daily functioning and quality of life.

Although the results offer important insights into the role of ergonomic practices and self-care measures in symptom management, the limitations of the study, including its cross-sectional design, reliance on self-reported data, and sample size, must be considered when interpreting the findings. Future research incorporating longitudinal designs, objective measures, and larger, more diverse samples would help validate these results and further explore the long-term effects of these interventions.

The study highlights the need to consider age-related differences in device access and usage patterns, as these factors may contribute to variations in posture and musculoskeletal symptoms. Tailored interventions for different age groups, focusing on their unique usage habits and needs, could enhance the effectiveness of strategies aimed at reducing symptom intensity and improving overall well-being.

Overall, this study emphasizes the need for proactive strategies in reducing musculoskeletal symptoms. Ergonomic tools, posture awareness, and regular breaks appear to be effective in managing symptoms, and further efforts to promote these practices could lead to improved health outcomes and productivity.

## Figures and Tables

**Table 1:** Descriptive statistics for participant age.

Variable	Age
Valid	222
Missing	0
Median	14.500
Mean	13.396
Std. Deviation	3.851
Range	12.000

**Table 2:** Participant demographics by gender, school level, and physical activity.

Variable	Level	Frequency	Percent
Gender	Boy	96	43.24%
	Girl	126	56.76%
School Level	Primary school	45	20.27%
	Middle school	67	30.18%
	High school	110	49.55%
Physical Activity	Active	52	23.42%
	Moderately active	59	26.57%
	Sedentary	111	50.00%
Missing		0	0.00%
Sample Size		222	100.00%

**Table 3:** Frequency of device usage among participants.

Variable	Level	Frequency	Percent
Smartphone	No	8	3.60%
	Yes	214	96.40%
Tablet	No	145	65.31%
	Yes	77	34.69%
Laptop	No	87	39.19%
	Yes	135	60.81%
Computer	No	132	59.46%
	Yes	90	40.54%
Sample Size		222	100.00

**Table 4:** Spearman’s correlation between device usage time and symptom intensity.

Variable		Device Usage Time	Symptom Intensity
Device Usage Time	Spearman’s rho	—	
	*p*-value	—	
Symptom Intensity	Spearman’s rho	0.908	—
	*p*-value	<0.001	—

**Table 5:** Model summary for regression analysis of symptom intensity.

Model	R	R^2^	Adjusted R^2^	RMSE
M_0_	0.000	0.000	0.000	2.181
M_1_	0.908	0.824	0.823	0.917

Note: M_0_ is the intercept-only model. M_1_ includes Device Usage Time.

**Table 6:** ANOVA results for the regression model predicting symptom intensity.

Model		Sum of Squares	df	Mean Square	F	*p*
M_1_	Regression	866.413	1	866.413	1029.845	<0.001
	Residual	185.087	220	0.841		
	Total	1051.500	221			

Notes: M_1_ includes Device Usage Time. The intercept model is omitted, as no meaningful information can be shown.

**Table 7:** Coefficients for the regression model predicting symptom intensity.

Model		Unstandardized	Standard Error	Standardized	t	*p*
M_0_	(Intercept)	7.500	0.146		51.231	<0.001
M_1_	(Intercept)	0.018	0.241		0.074	0.941
	Device Usage Time	1.890	0.059	0.908	32.091	<0.001

**Table 8:** Chi-squared test results for the association between posture and symptoms.

Variable	χ^2^	df	*p*
Neck Pain	267.915	16	<0.001
Shoulder Pain	299.668	16	<0.001
Back Pain	256.901	16	<0.001
Headaches	251.836	16	<0.001
Numbness	286.069	16	<0.001
Eye Strain	284.561	16	<0.001
Wrist Pain	251.436	16	<0.001
Symptom Intensity	269.741	32	<0.001
Sample Size	222		

**Table 9:** Symptom intensity distribution.

Variable	Level	Frequency	Percent
Neck Pain	1	4	1.80%
	2	24	10.81%
	3	38	17.12%
	4	70	31.53%
	5	86	38.74%
Shoulder Pain	1	5	2.25%
	2	21	9.46%
	3	34	15.32%
	4	69	31.08%
	5	93	41.89%
Back Pain	1	4	1.80%
	2	23	10.36%
	3	37	16.67%
	4	63	28.38%
	5	95	42.79%
Headaches	1	2	0.90%
	2	22	9.91%
	3	33	14.87%
	4	76	34.23%
	5	89	40.09%
Numbness	1	5	2.25%
	2	23	10.36%
	3	33	14.87%
	4	77	34.68%
	5	84	37.84%
Eye Strain	1	5	2.25%
	2	20	9.01%
	3	40	18.02%
	4	62	27.93%
	5	95	42.79%
Wrist Pain	1	6	2.70%
	2	20	9.01%
	3	35	15.77%
	4	77	34.68%
	5	84	37.84%
Symptom Intensity	1	0	0.00%
	2	1	0.45%
	3	11	4.95%
	4	18	8.11%
	5	22	9.91%
	6	16	7.21%
	7	16	7.21%
	8	49	22.07%
	9	42	18.92%
	10	47	21.17%
Sample Size		222	100.00%

**Table 10:** Chi-squared test of symptom intensity distribution by ergonomic tool usage.

Variable	χ^2^	df	*p*
Ergonomic Tools	306.803	32	<0.001
Sample Size	222		

**Table 11:** ANOVA for symptom intensity across ergonomic tool usage groups.

Cases	Sum of Squares	df	Mean Square	F	*p*
Ergonomic Tools	716.207	4	179.052	115.881	<0.001
Residuals	335.293	217	1.545		

**Table 12:** Rank correlation between break frequency and symptom intensity.

Variable		Symptom Intensity	Breaks
Symptom Intensity	Spearman’s rho	—	
	*p*-value	—	
Breaks	Spearman’s rho	0.908	—
	*p*-value	<0.001	—

**Table 13:** Chi-square test results for the association between posture awareness and symptom intensity.

Variable	χ^2^	df	*p*
Posture Awareness	180.547	24	<0.001
Sample Size	222		

**Table 14:** Relationship between measures taken to alleviate symptoms and their perceived effectiveness.

Variable	χ^2^	df	*p*
Effectiveness	343.855	16	<0.001
Sample Size	222		

**Table 15:** Chi-square test results for the impact of symptoms on daily activities.

Variable	χ^2^	df	*p*
Neck Pain	232.260	16	<0.001
Shoulder Pain	254.075	16	<0.001
Back Pain	261.429	16	<0.001
Headaches	246.721	16	<0.001
Numbness	244.467	16	<0.001
Eye Strain	281.664	16	<0.001
Wrist Pain	249.263	16	<0.001
Sample Size	222		

**Table 16:** Results for symptom intensity and activity avoidance.

Cases	Sum of Squares	df	Mean Square	F	*p*
Avoiding Activities	588.884	1	588.884	280.048	<0.001
Residuals	462.616	220	2.103		

**Table 17:** Cronbach’s α coefficient for the overall scale.

			95% CI	
Coefficient	Estimate	Standard Error	Lower	Upper
Coefficient α	0.965	0.002	0.961	0.968

**Table 18:** Cronbach’s α if Item Dropped.

	Coefficient α (If Item Dropped)		
Item	Estimate	Lower 95% CI	Upper 95% CI
Avoiding Activities	0.964	0.960	0.968
Back Pain	0.961	0.957	0.965
Breaks	0.962	0.958	0.966
Computer	0.967	0.964	0.971
Device Usage Time	0.960	0.956	0.965
Effectiveness	0.961	0.957	0.965
Ergonomic Tools	0.961	0.957	0.965
Eye Strain	0.961	0.957	0.965
Headaches	0.961	0.957	0.965
Impact on Activities	0.962	0.958	0.966
Laptop	0.968	0.965	0.972
Measures Taken	0.961	0.957	0.965
Neck Pain	0.961	0.957	0.965
Numbness	0.961	0.957	0.965
Posture Awareness	0.962	0.958	0.965
Posture	0.961	0.957	0.965
Shoulder Pain	0.961	0.957	0.965
Smartphone	0.967	0.963	0.971
Symptom Intensity	0.965	0.960	0.969
Tablet	0.967	0.964	0.971
Wrist Pain	0.961	0.957	0.965

## Data Availability

The raw data supporting the conclusions of this article will be made available by the authors upon request. The data are not publicly available due to ethical considerations and participant confidentiality.

## Supplementary Materials

The following supporting information can be downloaded at https://www.mdpi.com/article/10.3390/children12010102/s1, File S1: Tech Neck Syndrome Questionnaire.

## Abbreviations

The following abbreviations are used in this manuscript:

NMQ	The Nordic Musculoskeletal Questionnaire
VAS	The Visual Analog Scale
RMSE	Root Mean Square Error
df	degree of freedom
α	Alpha
CI	Confidence Interval
