# Supplementary material for: Evaluating Tech Neck: A Pilot Study Using a Self-Developed Questionnaire on Symptoms, Posture, and Preventive Measures
Source: Children (Basel). 2025 Jan 17;12(1):102. doi: 10.3390/children12010102 (PMC11764141; doi:10.3390/children12010102)
Supplement: Supplementary file 1 [file children-12-00102-s001.zip › children-3426930-supplementary.pdf]

Tech Neck Syndrome Questionnaire

Section S1: Demographic Data

1. **Age**: ___
2. **Gender**:
    - Boy
    - Girl
3. **School Level:**
    - Primary school (Grades 1-4)
    - Middle school (Grades 5-8)
    - High school (Grades 9-12)
4. **Physical Activity:**
    - I play a lot, exercise, or do sports often (Active)
    - I play or move around sometimes (Moderately active)
    - I spend most of my time sitting or relaxing (Sedentary)

Section S2: Device Usage

1. **Which of these do you use daily?**
    - Smartphone
        - No
        - Yes
    - Tablet
        - No
        - Yes
    - Laptop
        - No
        - Yes
    - Computer
        - No
        - Yes
2. **How long do you use these devices each day?**
    - Less than 1 hour
    - 1-3 hours
    - 3-5 hours
    - 5-8 hours
    - More than 8 hours
3. **How do you usually sit or lie when using your devices?**
    - Sitting at a desk
    - Lying in bed
    - Standing
    - Sitting on a couch
    - Other: ___
4. **Do you take breaks while using your devices?**
    - Yes, every 30 minutes

- Yes, every hour
- Rarely
- Never

Section S3: Symptoms and Associated Discomfort

1. **Have you felt any of these problems in the last 3 months?**

Symptom	Never (1)	Rarely (2)	Sometimes (3)	Often (4)	Always (5)
Neck pain	☐	☐	☐	☐	☐
Shoulder pain	☐	☐	☐	☐	☐
Back pain	☐	☐	☐	☐	☐
Headaches	☐	☐	☐	☐	☐
Numbness in hands/arms	☐	☐	☐	☐	☐
Eye strain (tired eyes)	☐	☐	☐	☐	☐
Wrist pain	☐	☐	☐	☐	☐

2. **How bad are these problems overall?** (Circle one number, where 1 means "very mild" and 10 means "very bad")
   1 2 3 4 5 6 7 8 9 10

Section S4: Ergonomics

1. **Do you use anything special to sit or study comfortably?**
   - Laptop stand
   - Special chair
   - Monitor stand
   - Other: ___
   - None

Section S5: Impact on Your Daily Life

1. **Do your neck or back problems stop you from doing things you like?**
   - Not at all
   - Not much
   - Yes, a little
   - Yes, a lot
2. **Have you ever skipped fun activities because of pain or discomfort?**
   - No
   - Yes

Section S6: What Do You Do to Feel Better?

1. **What do you do to feel better when your neck or back hurts?**
    - Get a massage or go to physical therapy
    - Use tools like a special chair or stand
    - Stretch my neck, back, or arms
    - Take breaks
    - Nothing
2. **Do these things help?**
    - Yes, a lot
    - Yes, a little
    - Not much
    - Not at all

Section S7: Posture Awareness

1. **How much do you think about your posture when using devices?**
    - ☐ I always think about it
    - ☐ I sometimes think about it
    - ☐ I rarely think about it
    - ☐ I don’t think about it at all
